# Protein Coding Gene Nucleotide Substitution Pattern in the Apicomplexan Protozoa *Cryptosporidium parvum* and *Cryptosporidium hominis*

**DOI:** 10.1155/2008/879023

**Published:** 2008-06-04

**Authors:** Guangtao Ge, Lenore Cowen, Xiaochuan Feng, Giovanni Widmer

**Affiliations:** ^1^Department of Computer Science, Tufts University, Medford, MA 02155, USA; ^2^Department of Biomedical Sciences, Tufts Cummings School of Veterinary Medicine, Tufts University, North Grafton, MA 01536, USA

## Abstract

*Cryptosporidium parvum* and *C. hominis* are related protozoan pathogens which infect the intestinal epithelium of humans and other vertebrates. To explore the evolution of these parasites, and identify genes under positive selection, we performed a pairwise whole-genome comparison between all orthologous protein coding genes in *C. parvum* and *C. hominis*. Genome-wide calculation of the ratio of nonsynonymous versus synonymous nucleotide substitutions (*d* *N*/*d* *S*) was performed to detect the impact of positive and purifying selection. Of 2465 pairs of orthologous genes, a total of 27 (1.1%) showed a high ratio of nonsynonymous substitutions, consistent with positive selection. A majority of these genes were annotated as hypothetical proteins. In addition, proteins with transmembrane and signal peptide domains are significantly more frequent in the high *d* *N*/*d* *S* group.

## 1. Introduction

Protozoan parasites belonging to the genus *Cryptosporidium* (Phylum: *Apicomplexa*) develop in the intestinal epithelium of many vertebrate species and frequently cause diarrhea. The two main species known to infect humans are *C.* *parvum* and *C. hominis*. Although highly similar at the genome level, these species differ in host range. Specifically, *C. parvum* infects humans and other mammals, particularly calves, but *C. hominis* is typically only found in humans. Human infections are mainly caused by the ingestion of drinking or recreational water contaminated with oocysts, an environmentally resistant form of the parasite.

The complete sequence of the *C. parvum* and *C. hominis*genomes was recently published [1, 2]. Both genomes are about 9 Mb in size and their gene complements appear to be identical, each coding for about 3900 genes. The *C. parvum* and *C. hominis* genomes exhibit only 3–5% sequence divergence and no large insertions or deletions have been identified [1]. Genome annotation has shown that energy metabolism pathways are present, but biosynthetic ability is limited. The pathways for de novo purine, pyrimidine, and amino acid synthesis are lacking. Consistent with the reliance of the intracellular developmental stages on the host cell for many metabolites, numerous genes encoding membrane transporters have been identified [1].

The analyses of nucleotide substitution patterns and genes under positive selection are of interest for understanding the evolution of these parasites and to improve genome annotation. Whereas “housekeeping genes” are under purifying selection to conserve many metabolic functions, genes that enable the parasite to adapt to different host environments may evolve more rapidly. Evolutionary analysis based on comparative genomics has been applied to different organisms, such as mammals [3–6], drosophila [7, 8], bacteria [9], and malaria [10, 11]. Here, we describe a genome-wide analysis of nucleotide substitution patterns in *C. parvum*/*C. hominis* orthologous protein coding genes.

## 2. Materials and methods

*C. parvum* and *C. hominis* protein coding gene nucleotide and amino acid sequences were downloaded from CryptoDB [12] (http://cryptodb.org/cryptodb/) release 3.2. There are 3806 genes from *C. parvum* and 3886 genes from *C. hominis* in total.

To identify orthologous genes between *C. parvum* and *C. hominis,* best reciprocal hits were first generated with BLASTP using an all-versus-all method and a significance threshold *e* < 10^−10^. The alignment regions were required to cover more than 80% of sequence lengths. This procedure resulted in 2465 pairs of orthologous gene sequences. To calculate *d* *N*/*d* *S*, amino acid sequences were first aligned using ClustalW1.83 [13] (http://www.ebi.ac.uk/clustalw/) with default parameters. Then, corresponding codon sequences were aligned based on the original protein sequence alignment using an in-house Perl script. The same process was also used on the raw ORF sequences to search for possible high *d* *N*/*d* *S* fragments that were erroneously annotated as being part of genes.

A PAML package implements three popular methods for calculating *d* *N*/*d* *S* values from sequence alignments [14] (http://abacus.gene.ucl.ac.uk/software/paml.html). These are a Nei and Gojobori method [15] based on a simple model of nucleotide substitution, and two more complex methods: ML94 and YN00 [16, 17]. ML94 method uses maximum likelihood framework to estimate *d* *N*/*d* *S* value directly based on codon substitution model, while YN00 method is another approximate counting method that incorporates the codon substitution model and unequal weight substitution pathways. We report results from the algorithm of Yang and Nielsen [16] that uses the codon model and nucleotide substitution model correction F84 and HKY85, and additionally weights pathways by their evolutionary probability. When we also calculated the *d* *N*/*d* *S* values using all three methods, the results were very similar. This is not surprising; results from the three methods are only expected to diverge significantly when the evolutionary distance between the organisms being compared is much larger.

Signal peptide motifs were predicted using SignalP v3.0 [18] at http://www.cbs.dtu.dk/services/SignalP/. Its neural network and hidden Markov model output YES or NO predictions individually. We considered one sequence to have a signal peptide if both, neural network and hidden Markov model, predicted positive results. Transmembrane domains were predicted using the TMHMM program v2.0 [19] at http://www.cbs.dtu.dk/services/TMHMM/. This program predicts the number of transmembrane domains and the number of amino acids in each domain. We considered any sequence to have a TM domain if at least one transmembrane helix included more than 18 amino acids. Protein functions were assigned based on sequence similarity search against NCBI nonredundant protein database.

To analyze the expression of selected genes by RT PCR, RNA was extracted from portions of the small intestine of an experimentally infected mouse. The gut was removed on day 14 post-infection from freshly euthanized animals and immediately chilled on ice. The epithelial cells were mechanically removed from the tissue. Total RNA was extracted from the cell scraping using Trizol reagent and further purified using a Qiagen RNA extraction kit. RNA was reverse transcribed into cDNA in the presence of poly-T primers. The absence of genomic DNA contamination from the RNA samples was confirmed by including an RNA control mock reverse transcribed in the absence of reverse transcriptase in each RT PCR experiment. We randomly selected 7 sequences from the high *d* *N*/*d* *S* (*d* *N*/*d* *S* > 1) group and 17 from the low *d* *N*/*d* *S* (*d* *N*/*d* *S* < 1) group. PCR primers for these sequences were designed using the LightCycler Probe Design software version 1.0 (Roche, Applied Science Indianapolis, IN.) with minimum cross complements. Amplifications were performed in a LightCycler (Roche Diagnostics) using FastStart SYBR Green I master mix (Roche Diagnostics). Initial denaturation was performed at 95°C for 10 minutes, followed by 40 cycles of amplification: denaturation at 95°C for 1 second, annealing at 64°C for 2 seconds, and extension at 72°C for 13 seconds. After amplification, melting curve analysis was performed as follows: initial denaturation at 95°C for 0 second, followed by annealing at 65°C for 15 seconds, and melting was performed in 0.1 degree increment per second until 95°C was reached for 0 second. After these procedures, amplification products were transferred from capillaries to microcentrifuge tubes, mixed with the loading buffer, and loaded onto a 1% agarose gel in a Tris-Acetate-EDTA buffer. Expression of selected sequences was confirmed if a single amplicon of the expected size was identified on ethidium bromide stained gels.

## 3. Results

**Figure 1:**
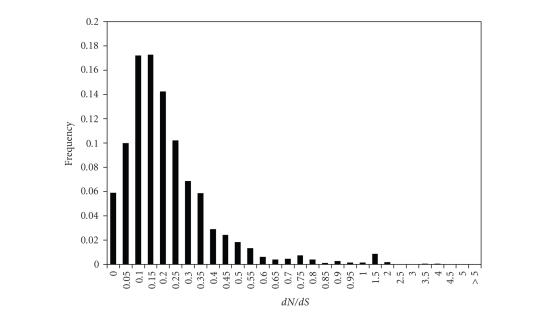
*d* *N*/*d* *S* ratio distribution of orthologous gene sequences from *C. parvum* and *C. hominis*. Black bars represent the frequency of gene sequences in each *d* *N*/*d* *S* category. For *d* *N*/*d* *S* < 1, category size is 0.05; for *d* *N*/*d* *S* > 1, category size is 0.5.

To identify the effect of selective pressure on the *Cryptosporidium* proteome, we started our analysis with 2465 pairs of orthologous *C. parvum* and *C. hominis* genes identified by BLASTP reciprocal best hits. Figures 1 and 2 in Supplementary Material, available online at doi:10.1155/2008/879023, show the *d* *S* and *d* *N* value distributions for *C. parvum* and *C. hominis*. Most of gene sequences have a *d* *S* ratio below 0.0625 and a *d* *N* ratio smaller than 0.025. Using the ratio of nonsynonymous over silent substitutions (*d* *N*/*d* *S*) [20], Figure 1 shows the distribution of *d* *N*/*d* *S* values among 2465 orthologous *C. parvum*/*C. hominis* gene pairs. As in two other whole-genome studies [3, 11], we found that most of the genes (2438 of 2465, 98.9%) are under negative selection (*d* *N*/*d* *S* < 1), while the remaining 1.1% evolve neutrally or have accelerated rates. This distribution is consistent with most annotated genes being genuine, as opposed to mispredictions or pseudogenes. The median *d* *N*/*d* *S* was 0.1484, similar to what was found in the taxonomically related malaria parasites [11, 21], where, for example, the median *d* *N*/*d* *S* values of 0.27, 0.30, and 0.17 for three pairwise comparisons between two laboratory isolates of *P. falciparum* and a *P. reichenowi* and a *P. falciparum* isolate from Ghana were found. Among the *C. parvum*/*C. hominis* orthologous gene pairs, 391 (15.8%) sequences had a *d* *N*/*d* *S* ratio less than 0.05. At the other extreme of the distribution, 27 (1.1%) genes with *d* *N*/*d* *S* > 1 were identified. This high *d* *N*/*d* *S* gene percentage is slightly lower than those of previous reports from human/chimpanzee orthologs comparison [6, 22].

To ensure that high *d* *N*/*d* *S* values did not originate from noncoding sequences erroneously annotated as genes, 7 *C. parvum* genes were randomly selected from 27 orthologs with *d* *N*/*d* *S* > 1 for RT-PCR analysis. Specifically, under the assumption that such genes were not a result of misannotation, we expected to detect mRNA transcripts from a similar ratio of genes with *d* *N*/*d* *S* > 1 as from genes with much lower *d* *N*/*d* *S* values. Thus, as controls, *d* *N*/*d* *S* transcripts with *d* *N*/*d* *S* < 1 were analyzed in parallel. For this analysis, *C. parvum* RNA was extracted from the intestinal epithelium of experimentally infected mice. Because such animal infections are not synchronized, we expected these RNA samples to contain transcripts of genes expressed at different stages of the intracellular life cycle. The RT PCR analysis successfully detected transcripts from 2 of 7 high *d* *N*/*d* *S* and 4 of 17 low *d* *N*/*d* *S* sequences (Table 1). The similar level of RT-PCR detection in sequences with high and low *d* *N*/*d* *S* indicates that high *d* *N*/*d* *S* genes are unlikely to have originated from the alignment of noncoding sequences erroneously identified as genes.

With genomes as close as *C. parvum* and *C. hominis*, there is always a possibility that high *d* *N*/*d* *S* values are found by chance. This is particularly a concern for the YN00 estimation method [16], which uses multiple correction terms for distant genomes. To verify our gene sets, we also ran the Nei and Gojobori NG86 method [15] and maximum likelihood method ML94 [17] on the data, which we would expect to show similar result with closely related genomes as is the case for *C. parvum* and *C. hominis*. In fact, for example, 22 of 27 gene pairs still exhibited *d* *N*/*d* *S* > 1 when ML94 method were used, whereas the remaining five all had *d* *N*/*d* *S* > 0.84.

**Figure 2:**
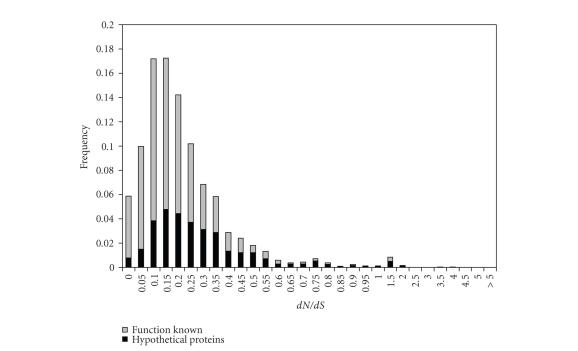
*d* *N*/*d* *S* ratio distribution of orthologous genes with or without functional annotation. Black bars represent gene with unknown function, while gray bars indicate known function.

For 797 (32.3%) of the 2465 orthologous gene pairs included in the *d* *N*/*d* *S* analysis the function is unknown. The remaining 67.7% (1668) have been assigned functional predictions based on homology to genes in other species at NCBI nonredundant protein database. Consistent with the difficulty of annotating fast-evolving genes, for high *d* *N*/*d* *S* genes (*d* *N*/*d* *S* > 1) putative functional annotation was rare: 59.2% of sequences with *d* *N*/*d* *S* > 1 (16 of 27) are annotated as “hypothetical protein.” Table 1 in supplementary material. In contrast, only 18.5% of sequences with *d* *N*/*d* *S* < 0.1 (151 of 814) are classified as hypothetical. The *d* *N*/*d* *S* distributions of these two groups of genes are significantly different (*P* < 6.51e-32, Komolgorov-Smirov (KS) test) as shown in Figure 2. The statistics of these distributions are summarized in Table 2.

**Figure 3:**
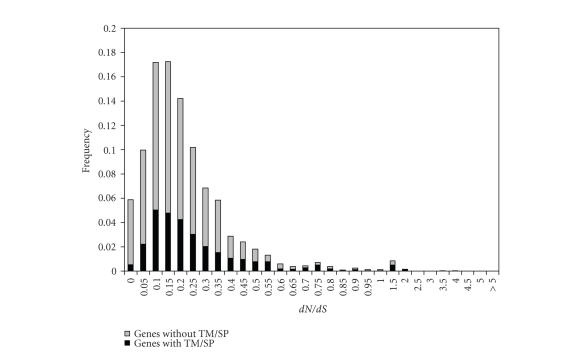
*d* *N*/*d* *S* ratio distribution of orthologous gene sequences with or without TM/SP structural domains. Black bars represent genes with TM/SP structural domain while gray bar represents the gene without TM/SP. TM: transmemebrane domain; SP: signal peptide.

Host-*Cryptosporidium* interaction is believed to involve many extracellular proteins with transmembrane (TM) domains or signal peptides (SP) [23]. Such proteins are likely to be exposed to the host immune response and may therefore evolve rapidly [24]. To investigate this hypothesis, we predicted TM and SP domains across all *C. parvum*/*C. hominis* orthologous gene pairs. A total of 16 of 27 sequences (59.2%) with *d* *N*/*d* *S* > 1 were predicted to have TM or SP domains, compared to 192 of 814 sequences (23.5%) with *d* *N*/*d* *S* < 0.1. We also observed that a higher percentage of sequences in the TM/SP group has *d* *N*/*d* *S* > 1 (2.2%), as compared to 0.6% of sequences without TM/SP. Figure 3 shows the *d* *N*/*d* *S* value distributions for these two groups. The two distributions are significantly different (*P* < 1.86e-7, KS test), consistent with many membrane proteins being under positive selection.

## 4. Discussion

The large proportion of *C. parvum* and *C. hominis* genes without functional annotation indicates the extent to which the genome of these parasites has been shaped by the adaptation to a complex life cycle, and to the host and outside environment. Bioinformatics contributes to our understanding of these genomes by flagging proteins showing signs of extreme selection. A computational approach is particularly relevant for studying pathogens such as *Cryptosporidium* species, which are difficult to culture, cannot be genetically manipulated, and are resistant to antiprotozoal drugs.

*Cryptosporidium* genomes are not intron-rich; only 5%–20% of genes are predicted to have multiple exons [1, 2]. Therefore, we also performed our *d* *N*/*d* *S* analysis using ORF sequences, so that we might find high *d* *N*/*d* *S* genes that were missed by the automatic gene annotation used in CryptoDB. We estimated selection pressures on *C. parvum* and *C. hominis* homologous ORF pairs using the same *d* *N*/*d* *S* statistics. A total of 251 out of 4279 orthologous ORFs (5.8%) showed signs of positive selection. One possible reason for the higher percentage is that some ORFs represent only parts of whole gene sequences, and averaging over multiple ORFs will lower *d* *N*/*d* *S* value of whole gene. For example, ORF CpIOWA_V_AAEE01000007-1-707587-707922 has a *d* *N*/*d* *S* value of 1.79, but further analysis showed that it is only a part of a protein which has a much lower *d* *N*/*d* *S* of 0.054. Nonetheless, it may be worth in future studies to examine these high *d* *N*/*d* *S* ORF regions more closely to elucidate if any high *d* *N*/*d* *S* genes were missed by gene finding methods.

Given the low divergence between *C. parvum* and *C. hominis*, estimation of *d* *N*/*d* *S* value may be inaccurate due to stochastic noise and a low level of substitutions. Most of the gene sequences have *d* *S* below 0.0625 (Supplemental Figure 1), which means that some of the high *d* *N*/*d* *S* values may be due to chance. Previous studies between closely related species such as human and chimpanzee showed that a large number of genes under positive selection are expected to occur by chance [6, 22]. The detection in our analyses of high *d* *N*/*d* *S* sequences with two different methods together with a significant enrichment for TM/SP sequences in these genes indicates that our analysis detects rapidly evolving genes.

As with other pathogens, sequences with TM/SP structures are expected to be directly involved in host-pathogen contact. In our study we identified a large proportion of sequences with predicted TM or SP motifs that have elevated *d* *N*/*d* *S* ratios. This observation is consistent with other studies. For instance, Jeffares et al. reported that *P. falciparum* genes mediating host cell invasion have high *d* *N*/*d* *S* ratios [11]. These authors also examined the substitution rates of genes according to cellular location. Significantly, more rapid rates of evolution were observed for predicted membrane-spanning proteins and exported proteins, as compared to proteins localized to the nucleus, cytoplasm, and mitochondrion. In another study comparing the genome of the rodent malaria species *P. berghei* and *P. chabaudi* [10], a significant difference between *d* *N*/*d* *S* values in TM/SP-containing and non-TM/SP-containing genes was found in proteins expressed during the blood stage, but not in genes expressed in the mosquito vector. A similar trend was also reported in a comparative study of the two *Theileria* species *T. annulata* and *T. parva* [25]. Whether similar life cycle dependent differences also occur in *Cryptosporidium* species or are a characteristic of *Apicomplexa* of the blood is an interesting topic for future investigation.

Even though many of the high *d* *N*/*d* *S* sequences in the *C. parvum*/*C. hominis* genome comparison remain without annotation, the few annotated genes in this category are worth mentioning. In particular, the genes with the second and sixth highest *d* *N*/*d* *S* values, (cgd2_440 with *d* *N*/*d* *S* > 3.36 and cgd2_220 with *d* *N*/*d* *S* > 1.5, resp.) are annotated as secreted mucins. Such mucins have been shown to be involved in attachment and invasion of host intenstinal epitethelial cells by *C. parvum* sporozoites and are crucially involved in pathogenesis of cryptosporidiosis [26]. They are found on the cell surface and apical region of invasive stages and are shed in trails during gliding motility [26, 27]. Another high *d* *N*/*d* *S* protein is from the DHHC family of palmitoyl transferases (cgd5_1260, with *d* *N*/*d* *S* > 1.46), which are enzymes involved in cellular signaling and membrane trafficking. In some parasitic protozoa palmatoylated proteins have been predicted to play a role in the evasion of the host immune response [28, 29].

## Supplementary Material

Supplemental Figure 1 describes the distribution of synonymous substitution ratios of genes calculated using the YN00 method. Substitution ratios show the number of synonymous substitutions over synonymous substitution sites. Supplemental Figure 2 illustrates the distribution of nonsynonymous substitution ratios of genes calculated as nonsynonymous substitutions over nonsynonymous substitution sites. Supplemental Table 1 shows a list of orthologous gene pairs showing signs of positive selection (dN/dS > 1; YN00 method). Values of dN/dS estimated from NG 86 and ML94 methods, as well as functional annotations, are also presented.

## Figures and Tables

**Table 1:** Summary of RT-PCR results.

	RT PCR detection	
	*d* *N*/*d* *S* > 1	*d* *N*/*d* *S* < 1
Total number of genes^(1)^	7	17
Number of detected	2	4
*d* *N*/*d* *S* (mean)	1.16	0.08
TM/SP (%)^(2)^	100	12
Annotated (%)^(3)^	28	88

^(1)^Number of transcripts which were detected or not detected by RT PCR ^(2)^Percent of transcripts predicted to encode a transmembrane domain or a signal peptide ^(3)^Percent of transcripts with annotated function.

**Table 2:** Summary of *d* *N*/*d* *S* analysis.

	Classification by structure		Classification by annotation	
	With TM/SP^(2)^	Without TM/SP	Hypothetical	Known function
Number of genes^(1)^	721	1744	797	1668
Median *d* *N*/*d* *S*	0.1762	0.1388	0.2124	0.1276
KS^(3)^ test *P* value	1.86e-7		6.51e-32	

^(1)^Number of orthologous gene sequences between *C. parvum* and *C. hominis* ^(2)^Transmembrane or signal peptide ^(3)^Kolmogorov-smirnov test.
