# Supplementary figures and images for: Protein Coding Gene Nucleotide Substitution Pattern in the Apicomplexan Protozoa Cryptosporidium parvum and Cryptosporidium hominis
Source: Comp Funct Genomics. 2008 Jun 4;2008:879023. doi: 10.1155/2008/879023 (PMC2413048; doi:10.1155/2008/879023)

## Slide 1
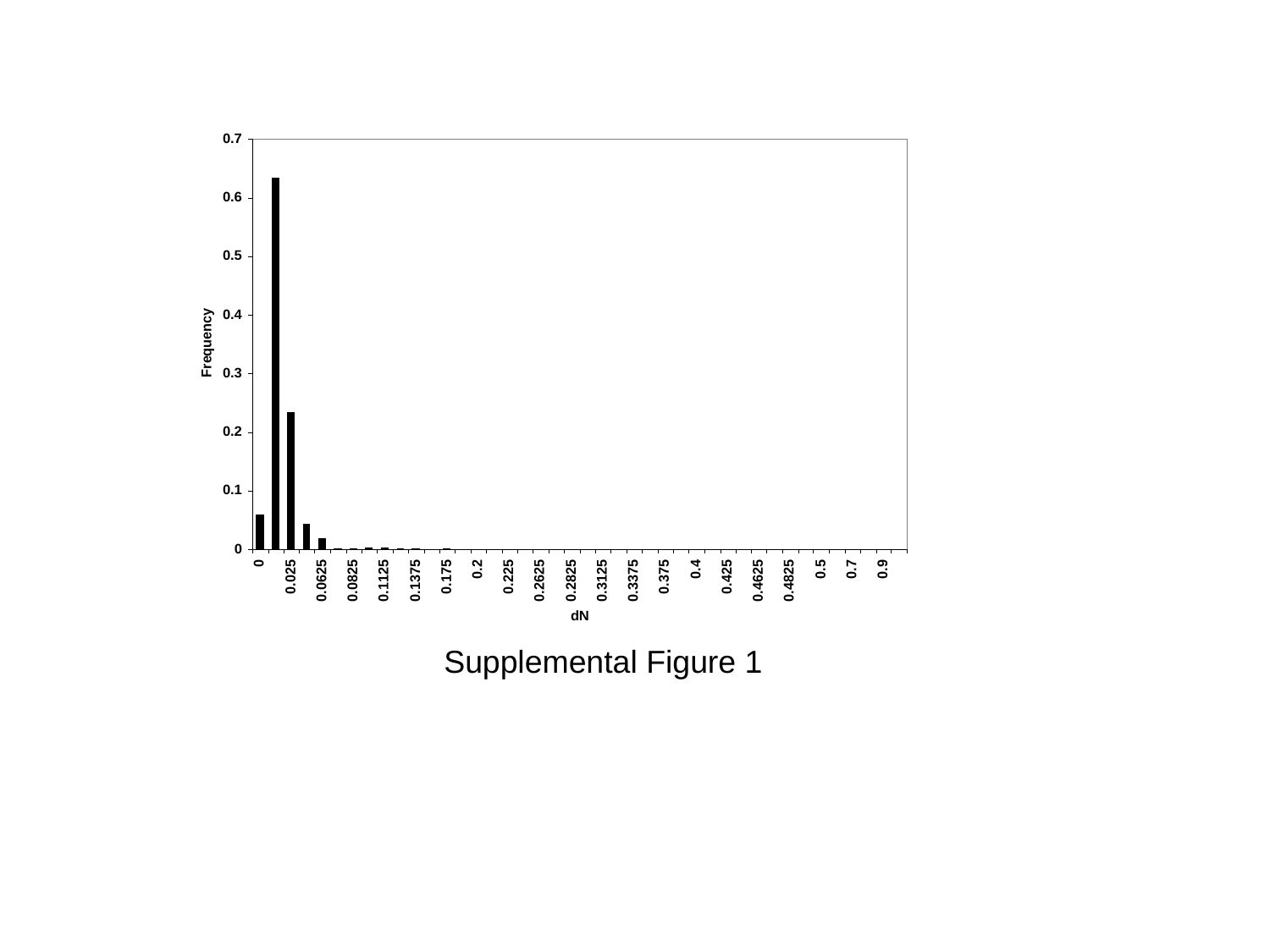

Supplemental Figure 1

Supplement: Supplementary file 1 — Supplemental Figure 1 describes the distribution of synonymous substitution ratios of genes calculated using the YN00 method. Substitution ratios show the number of synonymous substitutions over synonymous substitution sites. Supplemental Figure 2 illustrates the distribution of nonsynonymous substitution ratios of genes calculated as nonsynonymous substitutions over nonsynonymous substitution sites. Supplemental Table 1 shows a list of orthologous gene pairs showing signs of positive selection (dN/dS > 1; YN00 method). Values of dN/dS estimated from NG 86 and ML94 methods, as well as functional annotations, are also presented. [file 879023.f1.ppt]

## Slide 1
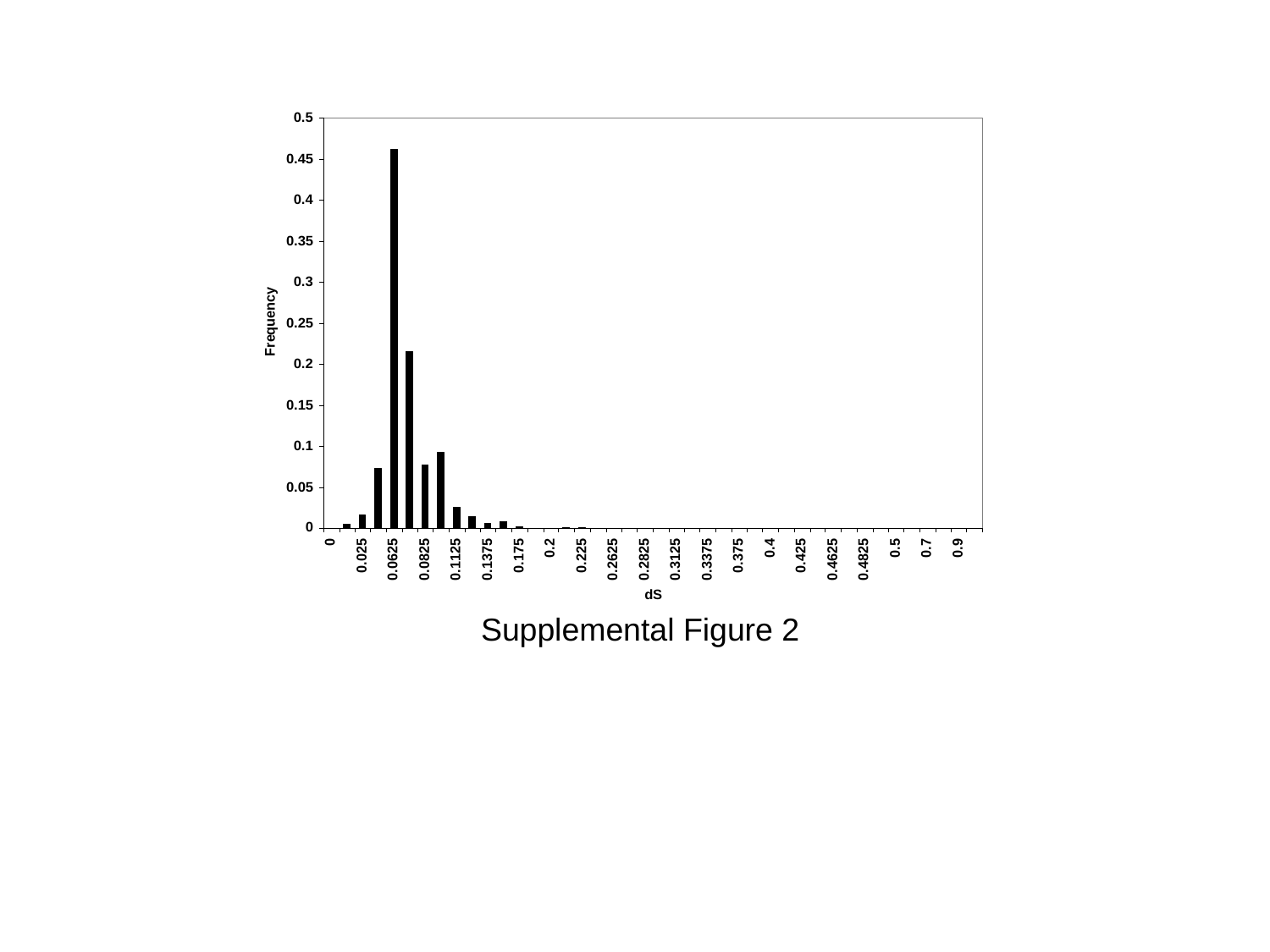

Supplemental Figure 2

Supplement: Supplementary file 2 [file 879023.f2.ppt]
